# Pyruvate kinase M2 regulates fibrosis development and progression by controlling glycine auxotrophy in myofibroblasts

**DOI:** 10.7150/thno.60385

**Published:** 2021-09-09

**Authors:** Ganesh Satyanarayana, Ravi Chakra Turaga, Malvika Sharma, Siming Wang, Falguni Mishra, Guangda Peng, Xiaonan Deng, Jenny Yang, Zhi-Ren Liu

**Affiliations:** 1 Department of Biology, Georgia State University, Atlanta, GA 30303, USA.; 2 Department of Chemistry, Georgia State University, Atlanta, GA 30303, USA.

**Keywords:** Pyruvate kinase M2, fibrosis, fibroblasts, collagen, Metabolism

## Abstract

**Rationale:** Fibrosis is a pathologic condition of abnormal accumulation of collagen fibrils. Collagen is a major extracellular matrix (ECM) protein synthesized and secreted by myofibroblasts, composing mainly (Gly-X-Y)n triplet repeats with >30% Gly residue. During fibrosis progression, myofibroblasts must upregulate glycine metabolism to meet the high demands of amino acids for collagen synthesis.

**Method:** Expression of PKM2 in myofibroblasts was analyzed in cultured fibroblasts and fibrosis disease tissues. Functional roles of PKM2 and PKM2 activator in biosynthesis of serine → glycine and production of collagen from glycolysis intermediates were assayed in cultured activated LX-2 and human primary lung fibroblast cells. Mouse models of Liver, lung, and pancreas fibrosis were employed to analyze treatment effects of PKM2 activator in organ tissue fibrosis.

**Results:** We report here that myofibroblast differentiation upregulates pyruvate kinase M2 (PKM2) and promotes dimerization of PKM2. Dimer PKM2 slows the flow rate of glycolysis and channels glycolytic intermediates to *de novo* glycine synthesis, which facilitates collagen synthesis and secretion in myofibroblasts. Our results show that PKM2 activator that converts PKM2 dimer to tetramer, inhibits fibrosis progression in mouse models of liver, lung, and pancreatic fibrosis. Furthermore, metabolism alteration by dimer PKM2 increases NADPH production, which consequently protects myofibroblasts from apoptosis.

**Conclusion:** Our study uncovers a novel role of PKM2 in tissue/organ fibrosis, suggesting a possible strategy for treatment of fibrotic diseases using PKM2 activator.

## Introduction

Collagen fibrils are excessively produced during fibrosis progression as a consequence of inflammation response to tissue damages 1, 2. Fibrosis affects tissues of almost all organs. Myofibroblasts are the main cell type that is engaged in collagen synthesis and secretion. Collagen is an ECM protein that is mainly composed of Gly-x-Pro and Gly-x-Hyp triplet repeats. Glycine constitutes >30% of the amino acids in collagen. Myofibroblasts must engage in excessive glycine production in order to meet the needs of massive collagen production and secretion during fibrosis progression. Cells obtain glycine from two sources: dietary source and *de novo* synthesis. Glycine obtained from diet is usually channeled into subsequent protein synthesis. However, during fibrosis progression, quick, easy, and massive glycine production route is through *de novo* biosynthesis 3.

Glycine *de novo* synthesis is dependent on glycolysis, wherein the rate of glycolytic intermediates channeled to ATP production is reduced, instead glycolytic intermediate 3-phosphoglycerate (3-PG) is channeled for serine and subsequent glycine production catalyzed by a series of four enzyme catalyzed reactions, 3-PG → 3-phosphohydroxypyruvate → 3-Phosphoserine (3-PS) → serine → glycine 4, 5. Clearly, synthesis of Gly is favored when the conversion of phosphoenolpyruvate (PEP) to pyruvate by pyruvate kinase is reduced. It is well established that converting PKM2 tetramer to dimer reduces glycolytic carbon flow to mitochondria for ATP production, which results in glycolytic intermediate accumulation 6, 7. It is reasonable to speculate that PKM2 may play a role in facilitating glycine synthesis during fibrosis progression. We present evidence to show here that PKM2 is upregulated in myofibroblasts. Myofibroblast differentiation promotes PKM2 dimer. PKM2 dimer slows the flow rate of glycolysis and channels glycolytic intermediates to *de novo* glycine synthesis, which facilitates collagen synthesis and secretion in myofibroblasts. The metabolism alteration by dimer PKM2 increases NADPH production, which consequently protect myofibroblasts from apoptosis. Survival of myofibroblasts facilitates fibrogenesis. Our experiments show that PKM2 activator inhibits fibrosis progression, suggesting a potential strategy of anti-fibrosis.

## Methods

All reagents, antibodies, cells (lines), kits, expression vectors, RNAi, and PCR primers used in this study are listed in tables in the on-line supplementary material.

### Liver and lung fibrosis mouse models and treatments

All animal experiments were carried out under approval of IACUC of Georgia State University.

#### Liver fibrosis and treatment

6 weeks old BALB/cJ mice were provided with 10% ethanol in drinking water *at libitum* and thioacetamide (TAA) was i.p. administered at 100 mg/Kg bi-weekly for 5 weeks. The dose was increased to 250 mg/Kg for another 7 weeks. Mice were treated with TEPP-46 at 50 mg/Kg i.p. daily for 21 days. Mice were euthanized and tissues were harvested for further analysis.

#### Lung fibrosis and treatment

6 weeks old C57BL/6J mice were i.p. administered with 4 mg/Kg bleomycin bi-weekly for 6 weeks. Mice were treated with TEPP-46 at 50 mg/Kg daily for 21 days. Mice were euthanized and tissues were harvested for further analysis.

#### Pancreatitis and treatment

6 weeks old C57BL/6 mice were injected with cerulein (50 µg/Kg i.p.) every hour for 6 hours. This regimen was followed for 6 days with cerulein injection every other day. TEPP-46 (50 mg/Kg) was then injected every day for 6 days. Mice were sacrificed at day 12 and the pancreas and other tissues were collected for analyses.

**Patient tissue** analyses were carried out in accordance with the guidelines of NIH. All tissue samples are de-identified. It falls under IRB exemption 4. Tissue samples were sectioned and analyzed by different staining. Samples were obtained from commercial sources.

#### Primary fibroblasts and activation

LX2 and NLF cells were purchased from Millipore (SCC064) and Lonza (CC-2512) respectively and cultured in complete fibroblast medium. The primary cells were activated by culturing in TGF-β (5 ng/ml) for 48 hours.

#### PKM2 multimeric state evaluation

LX2 cells were activated with TGF-β (5 ng/mL) for 48 hours. They were then treated with either vehicle (DMSO) or DASA-10 (10 mM) for 12 hours and collected in ice cold PBS. Similarly, liver tissues from mice treated with either vehicle or TEPP-46 were flash frozen and re-suspended in ice cold PBS. Both cells and tissue slurries were then incubated with 3 mM BS3 for 30 minutes in room temperature. They were then washed with ice cold PBS and were lysed. Crosslinked proteins were subjected to native gel electrophoresis followed by immunoblot for PKM2.

### HPLC and Mass spectrometry data quantitative analysis

1×10^6^ cells or 100 mg tissue samples were used for all metabolites and amino acids HPLC-ms analyses. Cells were washed with 0.9% NaCl and flash fixed in precooled 50% methanol. The cells were then scraped and transferred to an Eppendorf tube. Chloroform was added to the samples and were lysed for 30 minutes at 4 °C. The lysates were centrifuged at 14000 rpm for 10 minutes and the aqueous phase was used for amino acid analyses.

LC-MS analysis was carried out with a Sciex API3200 ESI-triple quandrupole mass spectrometer coupled with an Agilent 1200 HPLC. A Phenomenex Germini NX-C_18_ column (3 μm, 100x3 mm) was used with flow rate of 200 μL/min. Mobile phase was 1% ACN containing 0.1% HCOOH (mobile phase B) and 99% water containing 0.1% HCOOH (mobile phase A). The analysis was isocratic for 10 min. 5 μL of each standard and sample was injected into the system. The MS ion source used was ESI in a positive selective reaction mode (SRM) with the precursor/product ion pairs listed in the following table. The MS parameters used are as follows: Ion source (IS) voltage, 5400 v, ion source temperature 450C, collision energy 515v. Analyst 1.5.1 was used for data analysis.

### Tissue section staining

**Sirius red and Masson's trichrome** were performed using kits obtained from IHC WORLD by following the instructions of vendor.

**IHC and IF:** IHC and IF staining procedures were similar to those of previous reports 8.

Images were captured at various magnification lens aperture and indicated by scale bars in the images.

Quantitation of Sirius red, Masson's trichrome, IHC, and IF staining was carried out using ImageJ. Quantities are presented as percent (%) of positive stain area in each view field or fold changes by comparing to the controls (vehicle), unless otherwise specified in the figures and legends. All quantitation results were means of randomly selected 3 view fields per section, 5 sections per animal, and 6-10 mice per experimental group, unless otherwise specified in the figures and legends.

### Statistical calculations

Statistical analyses were carried out using the GraphPad Prism 6.0 software. All experiments were carried out in 5 times minimum. Statistical significance was assayed by either Student's t-test and/or one-way ANOVA for multiple comparisons followed by post-hoc Tukey's test. Box plots show range, median and quartiles. In all figures, *P < 0.05; **P < 0.01, ***P < 0.001, **** P < 0.0001; n.s. denotes not significant. All data are presented as mean ± s.e.m. or as box plots.

## Results

### Myofibroblast differentiation upregulates PKM2 dimer facilitating Gly metabolism

**Figure 1:**
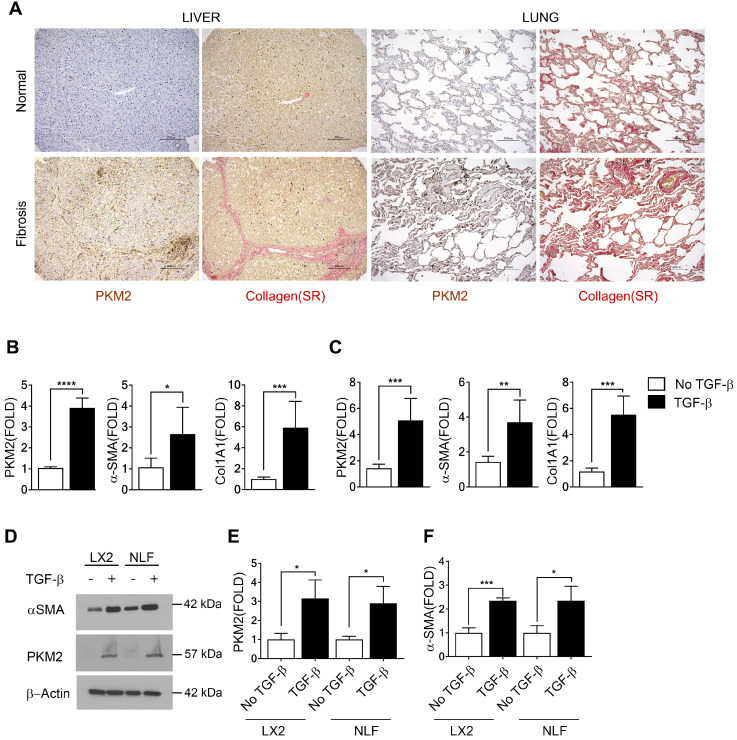
** PKM2 is elevated in myofibroblasts and activated hepatic stellate cells. (A)** Representative (Tissue samples from commercially available tissue arrays; liver fibrosis n = 40, normal liver n = 4, lung fibrosis n = 40, normal lung n = 3, were analyzed) images of IHC staining of PKM2 and Sirius red staining of collagen of normal (Upper) and liver (Bottom left) and lung (Bottom right) fibrosis patient samples. **(B & C)** Levels of PKM2, α-SMA, and collagen 1 (Col1A1) mRNA in LX-2 cells (B) and NLF (C) with (black bar) or without (open bar) TGFβ treatment (10 ng/mL for 48 hours) were analyzed by qRT-PCR. The mRNA levels were presented as fold changes by defining the mRNA level in the cells without TGFβ treatment as 1. **(D)** Levels of PKM2 (IB:PKM2) and α-SMA (IB:α-SMA) in LX-2 cells (LX2) and NLF with (+) or without (-) TGFβ treatment were analyzed by immunoblot. Immunoblot of β-actin (IB:β-actin) is a loading control. Molecular weights (MW) are indicated on the right*.* **(E & F)** Quantification of PKM2 (E) and α-SMA (F) levels in LX2 cells and NFL with (TGFβ, black bar) or without (no TGFβ, open bar) TGFβ treatment. The PKM2 and α-SMA levels are presented as fold changes by comparing to controls. Error bars in B, C, D, F represent mean ± S.E.M.

**Figure 2:**
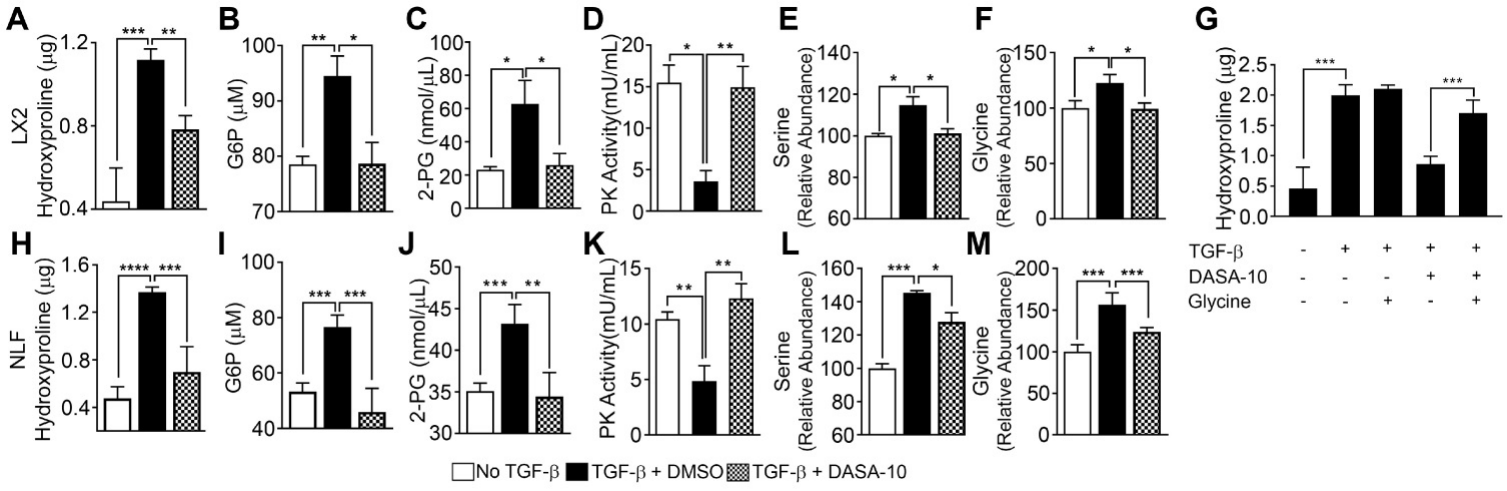
** TGFβ decreases pyruvate kinase activity and increases serine-glycine metabolism. PKM2 activator decreases serine-glycine metabolism in myofibroblasts. (A & H)** Hydroxyproline in LX2 (A) and NFL (H) with (filled bars) or without (open bars) TGFβ treatment and treated with DASA-10 (grey bars) after TGFβ treatment was analyzed using hydroxyproline kit. **(B, C, I & J)** Glycolytic intermediates G6P (B, I) and 2-PG (C, J) in LX2 (B, C) and NFL (I, J) with (black bar) or without (open bar) TGFβ treatment and treated with DASA-10 after TGFβ treatment (grey bar) were analyzed using commercial kits. The G6P is presented as μM (1x10^6^ cells lysate in 100 μL), 2-PG is presented as nmole per μL cell lysate. **(D, K)** Pyruvate kinase activity of lysate of LX2 (E) and NFL (J) with (black bar) or without (open bar) TGFβ treatment and treated with DASA-10 after TGFβ treatment (grey bar) was analyzed using pyruvate kinase activity kit. Pyruvate kinase activity is presented as mU/mL cell lysate. **(E, F, L & M)** Levels of serine (E, L) and glycine (F, M) in lysate of LX2 (E, F) and NFL (L, M) with (black bar) or without (open bar) TGFβ treatment and treated with DASA-10 after TGFβ treatment (grey bar) was analyzed using amino acid analysis kits. **(G)** Hydroxyproline in LX2 with or without TGFβ treatment and with or without DASA-10 treatment after TGFβ treatment (as indicated) was analyzed using hydroxyproline kit. The hydroxyproline in A, G, and H is presented as μg of hydroxyproline in lysate of 1x10^6^ cells. Error bars in all panels represent mean ± S.E.M.

We first probed expression of PKM2 protein in normal healthy human liver and lung tissues and compared to its expression in liver and lung fibrosis patient tissues. Evidently, PKM2 is strongly expressed in both fibrotic liver and lung tissues, while the protein is not expressed in normal healthy liver and lung tissue (Figure 1A). Co-IF staining of α-SMA and PKM2 protein in the sections from fibrotic liver of murine model revealed co-staining of α-SMA with PKM2, indicating PKM2 expression in the activated hepatic stellate cells (HSC) in fibrotic liver (Figure S1A). We then probed PKM2 expression in cultured fibroblasts in both mRNA and protein levels. PKM2 is almost not expressed in quiescent human lung fibroblasts (NLF) and inactivated HSC. Upon myofibroblast differentiation and activation of HSC by TGFβ, expression of PKM2 both mRNA and protein were upregulated in all myofibroblasts (Figure 1B-E). We confirmed the myofibroblast differentiation and stellate cell activation by analyzing cellular levels of α-SMA and collagen 1 both in mRNA and protein levels in the treated cells (Figure 1 B, C, D, and F). We then pursued to analyze if the glycolytic intermediates were indeed channeled to Gly synthesis and collagen production in the myofibroblasts. Human hepatic stellate LX2 cells and NLF were activated by TGFβ. Cellular levels of collagen were analyzed by immunoblot and hydroxyproline assay. Clearly, TGFβ increased the collagen mRNA and protein levels in the cells (Figure S1B, Figure 2A & H). Phosphoglycerate dehydrogenase (PHGDH) is the key enzyme that catalyzes the conversion from 3-phosphoglycerate (3PG) to serine and subsequently to glycine. Immunoblot analysis indicated that TGFβ increased cellular levels of PHGDH (Figure S1B & C), suggesting that the metabolic activity of converting the glycolytic intermediate 3PG to glycine is increased in myofibroblasts. Consistently, the glycolytic intermediates glucose-6-phosphate (G6P) and 2-phosphoglycerate (2-PG) were increased upon TGFβ treatment (Figure 2B, C, I, and J). We then analyzed the Ser and Gly levels in the treated and untreated cells. TGFβ increased serine and glycine in myofibroblasts (Figure 2E, F, L, and M). It is known that expression of PKM2 and conversion PKM2 tetramer to dimer reduces the flow rate of ATP production in glycolysis and leads to the accumulation of glycolytic intermediates for biosynthesis 9. Thus, we measured PKM2 tetramer and dimer status in myofibroblasts upon TGFβ treatment. Myofibroblast differentiation led to an increase in PKM2 dimer and a decrease in PKM2 tetramer (Figure S1D-G). Consistently, TGFβ treatment reduced pyruvate kinase activity and increased the accumulation of glycolytic intermediates in cells (Figure 2D & K). DASA-10 is a PKM2 activator that promotes conversion of PKM2 dimer to tetramer 10. DASA-10 treatment following TGFβ treatment of LX2 cells led to increase in PKM2 tetramer and decrease in PKM2 dimer in the myofibroblasts as demonstrated by both chromatography fractionation and chemical crosslinking followed by immunoblot assays (Figure S1D-G). Treatment of LX2 and NLF cells by TGFβ led to an increase in cellular glycine and collagen. Subsequent treatment of the activated HSC LX2 with DASA-10 led to a decrease in serine and glycine levels almost to that of inactivated LX2 (Figure 2E, F, L, and M). Consistently, myofibroblast differentiation and stellate cell activation by TGFβ followed by DASA-10 treatment led to an increase in pyruvate kinase activity (Figure 2D & K) and a decrease in accumulation of glycolytic intermediates, G6P and 2PG in these cells (Figure 2B, C, I, and J). TGFβ resulted in an increase in PHGDH and treatment of activated HSC LX2 with DASA-10 also led to a decrease in collagen (Figure S1B, Figure 2A & H). However, DASA-10 treatment did not lead to a change of cellular PHGDH (Figure S1E), suggesting that DASA-10 mediated reduction in serine and glycine levels is not due to PHGDH enzyme levels, but is due to the change in 3PG substrate concentration. Consistently, DASA-10 treatment of LX2 cells following TGFβ led to decreases in enzymes that are involved in serine and glycine metabolism (Figure S2). To further verify that PKM2 activator decreased glycine synthesis therefore reduced collagen expression, we added glycine into the culture of TGFβ and DASA-10 treated LX2 cells. Hydroxyproline assay demonstrated that addition of glycine rescued collagen production (Figure 2G). We conclude from our experiments that HSC activation and myofibroblast differentiation promote PKM2 expression and dimerization. Dimer PKM2 in myofibroblasts facilitates *de novo* glycine synthesis by increasing glycolytic intermediates in the cells.

### PKM2 dimer facilitates fibrosis progression in murine models

**Figure 3:**
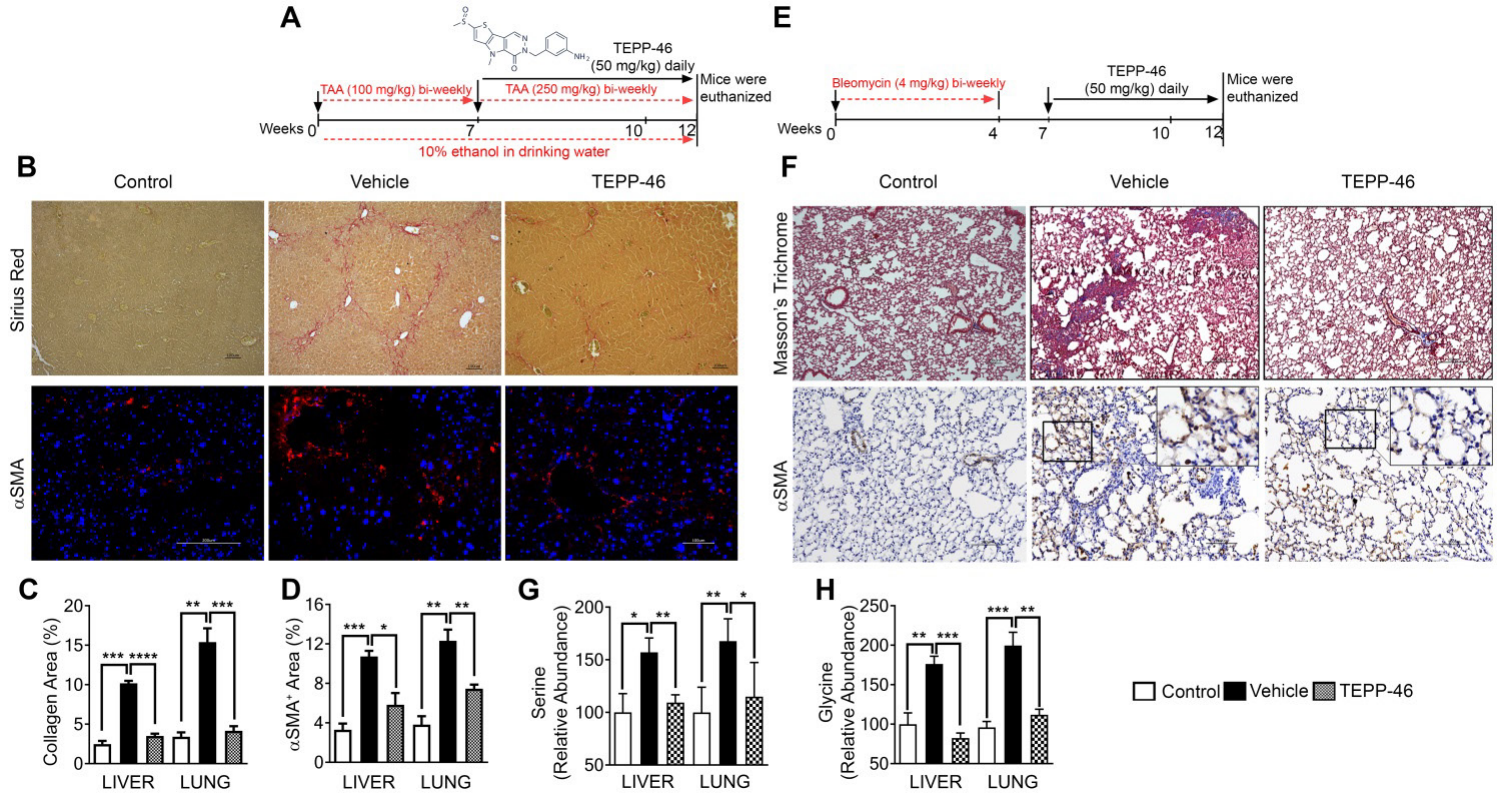
** TEPP46 reverses liver and lung fibrosis *and* decreases serine glycine metabolism in fibrotic liver and lung. (A & E)** Schematic illustration of the schedule of TAA/alcohol liver fibrosis (A) and bleomycin lung fibrosis (E) induction and subsequent TEPP46 treatments. **(B)** Representative images of Sirius red staining (Upper) and α-SMA IF staining (Bottom) of liver sections from mice treated with indicated agents. **(C & D)** Quantitation of collagen levels in Sirius red (B) and Masson's trichrome (F) staining and α-SMA IF (B) and IHC (F) staining of fibrotic liver (B) and lung (F) using ImageJ software. Quantification was calculated from measurements of 10 mice. Four randomly selected tissue sections per animal and three randomly selected view fields in each section were quantified. The quantity of collagen levels in Sirius red or Masson's trichrome stain and α-SMA IF or IHC stain is presented as % of total area. **(F)** Representative images of Masson's trichrome staining (Upper) and α-SMA IHC staining (Bottom) of lung tissue sections from mice treated with indicated agents. **(G & H)**, Quantitative analyses of Serine (G) and Glycine (H) levels in tissue extracts of liver and lung from mice treated with indicated agents by HPLC-MS. Control is the amino acid levels in normal liver and lung without fibrosis and treatment. Serine and glycine levels are presented as relative abundance by defining the vehicle treated group as 100%. Error bars in C, D, G, H represent mean ± S.E.M.

To test whether PKM2 expression and dimerization in myofibroblasts indeed regulate glycine metabolism and subsequent collagen synthesis and secretion *in vivo*, we employed a mouse model of liver fibrosis. Liver fibrosis was induced by administration of thioacetamide (TAA) + 10% ethanol in drinking water 11, 12. It is reported that DASA-10 is an PKM2 activator with excellent *in vitro* activity, while another PKM2 activator TEPP46 is more effective *in vivo* 10. Thus, we chose TEPP46 in our *in vivo* experiments. After fibrosis induction, the animals were treated with TEPP46 or vehicle (Figure 3A). TEPP46 treatment led to reduced fibrotic features on the surface of the liver (Figure S3A) and an increase in body weight compared to those of vehicle treated group (Figure S3B). Examination of serum markers of liver damage showed elevated levels of AST and ALT in vehicle treated mice and these were lowered significantly in TEPP46 treated mice (Figure S3C & D). Analysis of hepatic fibrosis with Sirius red staining confirmed that the mice that were treated with vehicle exhibited significant collagen deposition within the liver parenchyma, whereas in TEPP46 treated mice, the liver sections demonstrated fewer and thinner collagen depositions, especially the dense and continuous collagen networks disappeared (Figure 3B & C). Hydroxyproline assay supported the reduction of collagen upon TEPP46 treatment (Figure S3E). IHC staining of α-SMA demonstrated a reduction of α-SMA positive cells in the fibrotic liver in TEPP46 treated animals compared to the vehicle treated animals (Figure 3B & D). These experiments suggest that TEPP46 effectively reverses liver fibrosis. To test whether PKM2 activator affect the glycine metabolism in the fibrotic liver, amino acid contents in the liver extracts prepared from the TEPP46 and vehicle treated animals were measured by LC-MS. TEPP46 significantly decreased serine and glycine levels in liver of TEPP46 treated animals compared to that of vehicle treated animals (Figure 3G & H). TEPP46 treatment had fewer effects on the levels of other amino acids in the liver (Figure S3F). To verify whether PKM2 activator promoted formation of PKM2 tetramer in liver of treated mice, PKM2 tetramer in liver lysate of the treated mice was analyzed by crosslinking followed by immunoblot. Evidently, TEPP46 increased PKM2 tetramer in the liver of treated mice (Figure S3G).

**Figure 4:**
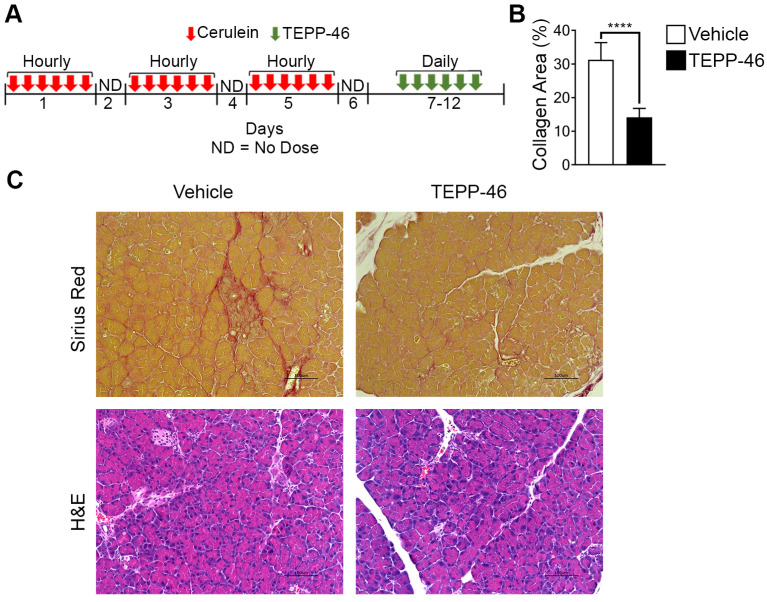
** PKM2 activator reverses acute pancreatitis. (A**) Schematic illustration of the schedule of cerulein (red arrows) pancreatitis induction and subsequent TEPP-46 treatments (green arrows). **(B)** Quantitation of collagen levels in Sirius red staining in (C) using ImageJ software. Quantification was calculated from measurements of 6 mice. Four randomly selected tissue sections per animal and three randomly selected view fields in each section were quantified. The quantity of collagen levels in Sirius red stain is presented as % of total area. **(C)** Representative images of Sirius red staining of collagen (Upper) and H&E staining of sections of pancreas from both vehicle and TEPP-46 treated animals. Error bars in B represent mean ± S.E.M.

High demand for the synthesis and secretion of collagen by myofibroblasts is a common phenomenon in all fibrosis diseases. To further verify the commonality of the function of PKM2 dimerization in glycine metabolism and subsequent collagen synthesis, we employed the bleomycin induce lung fibrosis model 13. Lung fibrosis was induced by i.p. administration of bleomycin twice weekly. These mice were treated with TEPP46 or vehicle by daily i.p. injection after the fibrosis induction by bleomycin (Figure 3E). At the end of the treatment, lungs from experimental animals were examined. Glossy examination of mouse lung indicated that TEPP46 led to less fibrotic features on the lung surface (Figure S3H) and reduced lung weight (Figure S3I) compared to the vehicle treated group. Masson's trichrome staining and hydroxyproline assay demonstrated that mice that were treated with vehicle exhibited significant collagen deposition in the lung, whereas TEPP46 led to thinner collagen deposition (Figure 3F & C, Figure S3J). IHC staining of α-SMA demonstrated a reduction of α-SMA positive cells in the fibrotic lung in TEPP46 treated animals compared to the vehicle treated animals (Figure 3G & D). Similarly, we analyzed the effects of TEPP46 on glycine metabolism in the bleomycin induced fibrotic lungs. Clearly, TEPP46 resulted in lower levels of serine and glycine in the extracts of lung of TEPP46 treated animals compared to the vehicle treated group (Figure 3G & H). Similarly, no significant effects of TEPP46 on other amino acid levels were observed in the lung fibrosis model (Figure S3J). Thus, experiments with both liver and lung fibrosis mouse models suggest that PKM2 activator reverses fibrosis by facilitating glycolysis flow, which leads to a decreased accumulation of glycolytic intermediates that are channeled to glycine synthesis. Reduced glycine metabolism limits collagen synthesis therefore reverses fibrosis progression. We further tested the effects of PKM2 activator in a cerulein induced acute pancreatitis mouse model (Figure 4A). Pancreatitis induction by cerulein clearly induced anatomic changes and collagen accumulation in pancreas of the mouse (Figure 4C). TEPP46 reduced collagen deposition in the pancreas of the treated animals and restored the anatomic structure of pancreas (Figure 4B & C). These results suggest that the PKM2 activator is effective in reversing cerulein induced pancreatic fibrosis. Treatment with TEPP46 in multiple organ models of fibrosis shows a global reduction in serine and glycine levels leading to decreased collagen production and secretion thereby slowing the progression of fibrosis.

### PKM2 dimer protects myofibroblasts from apoptosis by facilitating NADPH production

**Figure 5:**
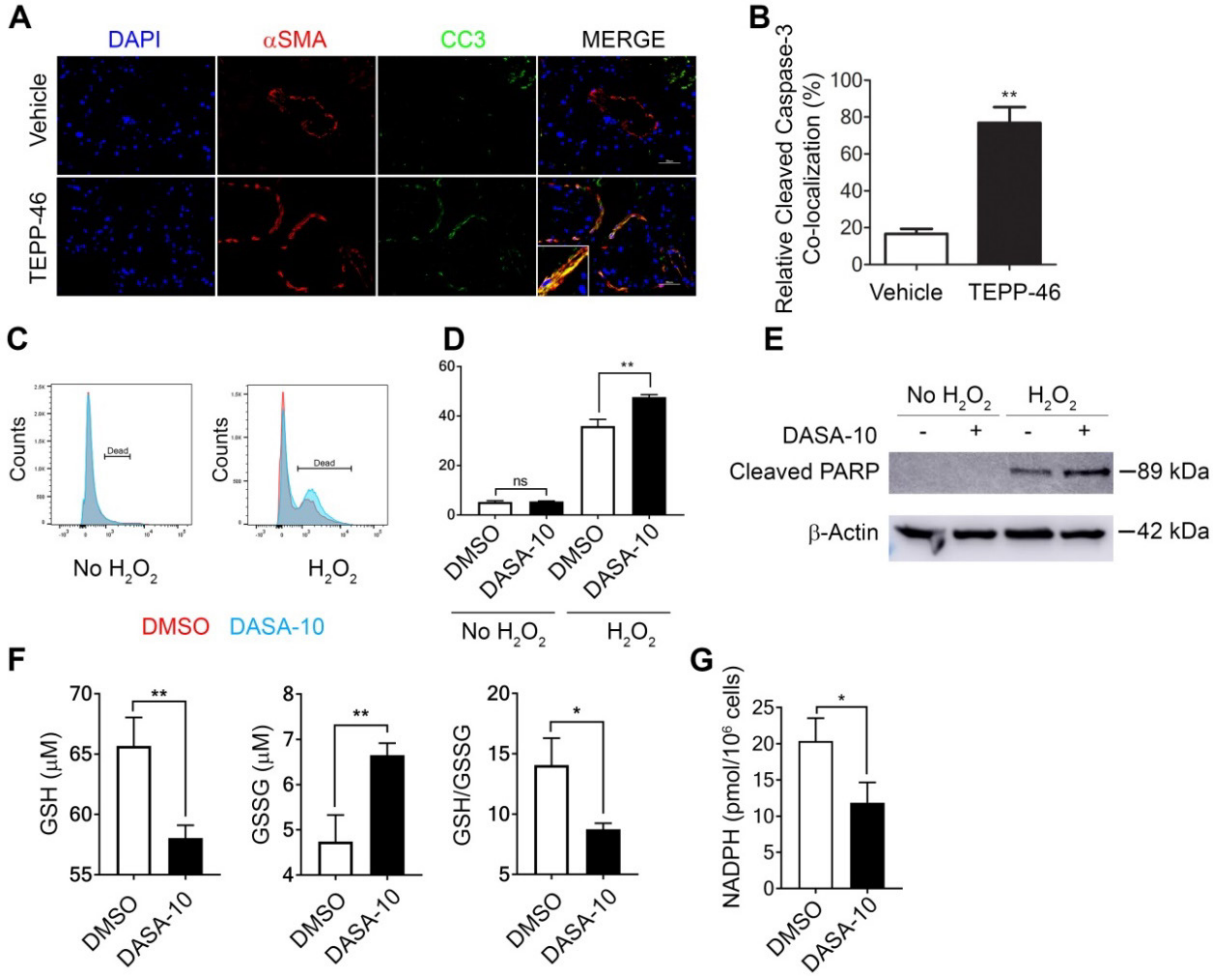
** Dimer PKM2 protects myofibroblasts from oxidative stress induced apoptosis by upregulation of NADPH metabolism. (A)** Representative images of IF co-staining of α-SMA with cleaved caspase 3 (CC3) of liver sections from mice treated with indicated agents.** (B)** Quantification of co-localization of cleaved caspase 3 positive cells and αSMA positive fibroblasts. **(C)** & **(D)** Apoptosis of LX2 with (H_2_O_2_) or without (no H_2_O_2_) treatment and with DASA-10 or DMSO treatment was measured by FACS (graph, **C**) and (quantitation, **D**). **(E)** Apoptosis in LX2 cells treated with or without H_2_O_2_ was analyzed by immunoblotting for cleaved PARP. **(F)** Cellular levels of GSH (Left panel), GSSG (Middle panel), ratio GSH/GSSG (Right panel) and **(G)** Total NADPH in LX2 treated with either DMSO or DASA-10 were measured using kits. In E, the MW is indicated on right. The cells were treated with either DMSO or DASA-10. GSH and GSSG are presented as μM (1x10^6^ cells lysate in 100 μL). NADPH is presented as pmole per 10^6^ cells. Error bars in C, D, and E represent mean ± S.E.M.

Interestingly, a decrease in α-SMA positive cells in liver and lung of the TEPP46 treated animals was observed (see Figure 3B & D and F & D). It is intriguing how TEPP46 reduces α-SMA positive HSC and myofibroblasts in the fibrotic liver and lung respectively. We sought to explore a possible molecular mechanism by which the PKM2 activator led to a reduction in myofibroblasts in these tissues. Our speculation is that dimer PKM2 protects myofibroblasts from apoptosis. Indeed, co-staining of α-SMA and cleaved caspase 3 (CC3) in the liver sections from TEPP46 and vehicle treated liver fibrosis animals showed that TEPP46 increased α-SMA and CC3 co-stains (Figure 5A & B). Fibrosis is caused by persistent inflammation. Persistent inflammation often brings high oxidative stress for surrounding cells, which frequently induces apoptosis of cells 14, 15. Reactive oxygen species (ROS) are elevated under oxidative stress to trigger apoptosis. We examined apoptosis of LX2 cells that were first treated by TGFβ and subsequently treated by DASA-10 or vehicle with or without H_2_O_2_. DASA-10 had almost no effect on TGFβ activated LX2 apoptosis without H_2_O_2_, while DASA-10 largely increased apoptosis of the LX2 cells in the presence of H_2_O_2_ (Figure 5C & D). Effects of DASA-10 on cell apoptosis were further analyzed by immunoblot of cleaved PARP. Clearly, DASA-10 increased apoptosis of the LX2 cells in the presence of H_2_O_2_ (Figure 5E). Glutathione (GSH) is the most abundant anti-oxidant in mammalian tissues and cells to handle oxidative stress. We therefore examined the GSH and oxidized GSSG in TGFβ activated fibroblasts with or without DASA-10 treatment. Clearly, DASA-10 treatment led to decreased GSH, increased GSSG, and decreased GSH/GSSG ratio (Figure 5F). The main pathway for production of the reducing power is pentose phosphate pathway (PPP). When glycolysis flow rate is reduced by dimer PKM2, production of reducing power NADPH by PPP is increased 6. The glycolytic intermediate G6P is the branch point to channel glucose to the PPP pathway. Evidently, fibroblast activation by TGFβ increased G6P (Figure 2C), and DASA-10 treatment decreased the intermediate G6P (Figure 3A). The consequence was a reduction in NADPH production upon DASA-10 treatment (Figure 5G). The lowered NADPH levels led to a decrease in GSH thereby lowering its anti-oxidant potential consequently leading to apoptosis.

### PTB regulates PKM splicing in myofibroblasts

**Figure 6:**
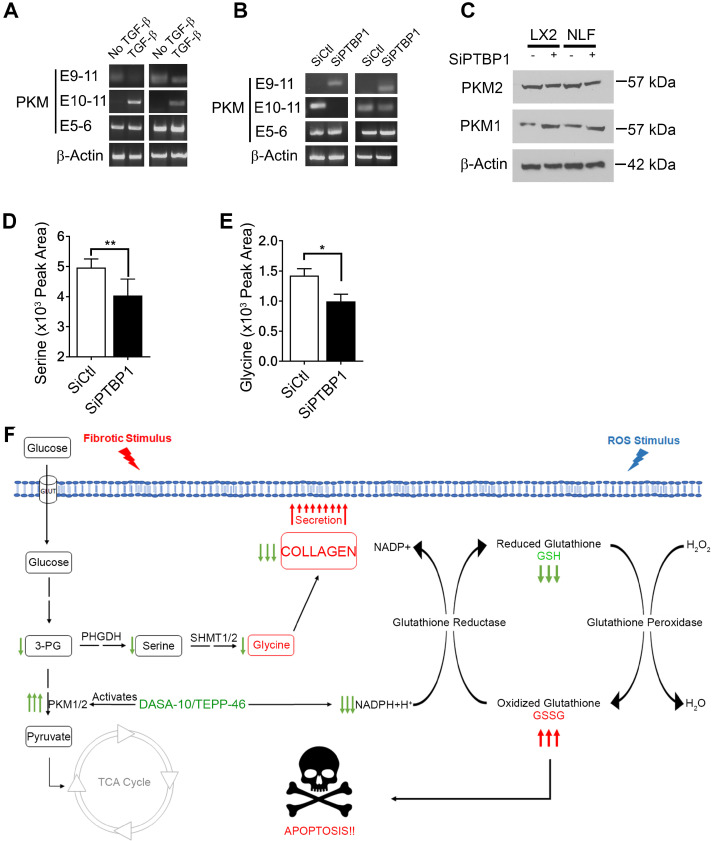
** PTB regulates PKM2 alternative splicing in myofibroblasts. (A & B)** Levels of mRNAs with E9-E11 (PKM2) or E10-E11 (PKM1) of PKM gene in LX2 cells and human primary lung fibroblasts (NLF) with (TGFβ) or without (no TGFβ) TGFβ treatment (A) or with (SiPTBP1) or without (Sictrl) PTB knockdown (B) was analyzed by qRT-PCR. E5-E6 is constitutive spliced mRNA of PKM gene as a control. qRT-PCR analysis of mRNA levels of β-actin is a loading control. **(C)** Cellular levels of PKM2 (IB:PKM2) and PKM1 (IB:PKM1) in LX2 cells and human primary lung fibroblasts (NLF) with (SiPTBP1) or without (Sictrl) PTB knockdown was analyzed by immunoblot. Immunoblot of β-actin (IB: β-actin) is a loading control. MW are indicated on the right. **(D & E)** Cellular levels of serine (D) and glycine (E) in lysate of LX2 cells with (SiPTBP1) or without (Sictrl) PTB knockdown was analyzed using amino acid analysis kits. **(F)** Carton illustrates the functions of PKM2 in facilitating fibrosis progression by acting in collagen metabolism and protecting myofibroblast from apoptosis. Error bars in D and E represent mean ± S.E.M.

We next sought to unravel the molecular mechanism by which activation of fibroblast regulates PKM2 expression. It is known that PKM2 expression in cancer cells is regulated by alternative splicing of the PKM gene 16, 17. We investigated whether myofibroblast differentiation by TGFβ also regulated PKM gene alternative splicing. LX2 and NLF were activated by TGFβ and the splice variants of the PKM gene in the cells was probed by RT-PCR. Clearly, TGFβ led to the production of PKM2 mRNA in both cells (Figure 6A). PTB plays a role in regulating PKM alternative splicing in cancer cells 18. We therefore also probed whether PTB regulated PKM alternative splicing in myofibroblasts. To this end, PTB was knocked down in LX2 and NLF. The cells were then activated by TGFβ. Examination of PKM splicing pattern by RT-PCR demonstrated that knockdown of PTB abrogated the effects of TGFβ in regulation of alternative splicing of PKM gene in the cells (Figure 6B & C). Myofibroblast differentiation regulates serine and glycine metabolism to facilitate collagen synthesis. If PTB plays a role in controlling PKM alternative splicing for production of PKM2, we would expect that PTB may play a role in regulation of serine and glycine metabolism. Indeed, knockdown of PTB lowered serine and glycine levels in TGFβ activated myofibroblasts (Figure 6D & E). We conclude from our studies that myofibroblast differentiation facilitates alternative splicing of the PKM gene to produce PKM2 by upregulation of PTB expression, which subsequently regulates synthesis of serine and glycine in the cells.

## Discussion

Glycolysis is interconnected with several metabolic pathways that coordinate catabolism and anabolism. Metabolites in glycolysis are often directly used in the synthesis of biomolecules, which are further utilized to synthesize protein, lipids, and nucleic acids to meet physiological or pathological needs of the cells. Myofibroblast is the sole source of large amounts of collagen secretion and deposition during fibrogenesis. Unique amino acid composition of the collagen requires rapid production of glycine and proline in large quantities in myofibroblasts during fibrogenesis. Upregulation of *de novo* glycine biosynthesis is most feasible route to meet the needs. Channeling glycolytic intermediates to serine synthesis pathway leads to *de novo* glycine biosynthesis. Thus, to meet the metabolic demand, myofibroblasts need to reduce glycolytic flow into mitochondrial TCA cycle and utilize the accumulating intermediates for anabolic processes. PKM2, particularly the dimer, is known to have reduced pyruvate kinase activity 19. Therefore, it is reasonable that myofibroblasts switch to PKM2 dimer in the cells, which may play a role to meet the needs of glycine and subsequent collagen synthesis (Figure 6F).

Consistent with our observation, Ding, H. and co-workers 20 and Yin, X. N. and co-workers 21 observed that PKM2 is upregulated in myofibroblasts of kidney fibrosis unilateral ureteral obstruction (UUO) mouse models and rat kidney NRK-49F cells upon TGFβ stimulation. Inactivation of pyruvate kinase activity and increase in aerobic glycolysis facilitates renal interstitial fibroblast activation and promotes renal fibrosis. Conversely, inhibition of aerobic glycolysis and activation of pyruvate kinase activity of PKM2 suppress renal fibrosis and fibroblast activation, the phenomenon similar to Warburg effect observed in cancer cells. These reports observed that PKM2 is expressed and inactivated, and as a consequence lactate is produced, in renal myofibroblasts. It is well documented that inactivation of PKM2 leads to an accumulation of glycolytic intermediates in cancer cells to favor biosynthesis. In order to maintain ATP and lactate production, cancer cells make-up glycolysis deficiency due to inactivation of PKM2 and pyruvate dehydrogenase by massive increase up-take of glucose (20-100 folds) 22-25. It is not known whether myofibroblasts also increase glucose up-take to maintain ATP and lactate production under condition of aerobic glycolysis and PKM2 inactivation.

Expression of PKM gene to PKM1 or PKM2 is controlled by alternative splicing with inclusion of exon 9 or exon 10 respectively 16, 17. In cancer cells, c-myc regulates PKM1/PKM2 splicing 17. It has been shown that PTB is involved in PKM gene alternative splicing regulation in cancer cells 18. Our results here show that PTB is also involved in PKM alternative splicing in myofibroblasts. The open question is how PTB is regulated in myofibroblasts. C-myc is upregulated in myofibroblasts 26. It is shown that PTB is upregulated by c-myc in cancer cells 16. Furthermore, the role of PTB in PKM splicing is controlled by c-myc 17. Thus, one possible mechanism is that TGFβ induces myofibroblast differentiation, which regulates c-myc and PTB expression. How TGFβ signaling promotes PKM2 dimer in myofibroblasts is another intriguing question. Growth signaling activates downstream tyrosine kinases, which subsequently leads to tyrosine phosphorylation of PKM2 promotes PKM2 tetramer to dimer conversion in cancer cells 27, 28. A number of tyrosine kinases are downstream targets of TGFβ signaling during myofibroblast differentiation 29, 30. Thus, it is plausible that tyrosine kinases activated by TGFβ in myofibroblasts may play a role in converting and maintaining PKM2 in its dimer state.

It is intriguing that PKM2 dimer not only channels glycolytic intermediates to glycine synthesis in myofibroblasts but also protects myofibroblasts from ROS mediated apoptosis. Survival of myofibroblasts sustains collagen production, a typical feature of fibrosis. This is consistent with our observation that PKM2 is elevated in tissue samples of liver and lung fibrosis patients. Dimer PKM2 mediated glycolytic switch from catabolism to anabolism fulfills the role of NAPDH production via PPP and subsequent other reductants, consequently, protecting myofibroblasts from oxidative stress induced apoptosis (Figure 6F). The dual roles of PKM2 in fibrosis progression make PKM2 an attractive target in anti-fibrosis therapy. PKM2 activator, which converts PKM2 dimer to tetramer, is indeed an effective approach for the treatment of liver, lung, and pancreatic fibrosis.

## Supplementary Material

Supplementary figures and table.
