# Supplementary material for: Pyruvate kinase M2 regulates fibrosis development and progression by controlling glycine auxotrophy in myofibroblasts
Source: Theranostics. 2021 Sep 9;11(19):9331–41. doi: 10.7150/thno.60385 (PMC8490528; doi:10.7150/thno.60385)
Supplement: Supplementary file 1 — Supplementary figures and table. [file thnov11p9331s1.pdf]

On-line supplementary

Pyruvate Kinase M2 Regulates Fibrosis Development and Progression by Controlling Glycine Auxotrophy in Myofibroblasts

Ganesh Satyanarayana[1], Ravi Chakra Turaga[1], Malvika Sharma[1], Siming Wang[2], Falguni Mishra[1], Guangda Peng[1], Xiaonan Deng[2], Jenny Yang[2], and Zhi-Ren Liu[1*]

[1]Department of Biology, [2]Department of Chemistry

Georgia State University, Atlanta, GA 30303, USA

Table 1. Cell lines, antibodies, reagents, PCR primers, and kits used in the study

**Cell lines**	**Company**	**Cat No.**
LX2	Millipore	SCC064
NLF Cells	Lonza	CC-2512
**Antibodies**	**Company**	**Cat No.**
Pkm1	Cell Signaling	7067s
Pkm2	Cell Signaling	4053s
Phgdh	Cell Signaling	66350s
Asma	Sigma	A5228
Actin	Yurogen	R15006MC4H
Anti Rabbit IgG	Thermo	31460
Anti Mouse IgG	Thermo	31430
**Reagents and kits**		
**Product**	**Company**	**Catalog number**
Glycine Kit	Abcam	AB211100
2PG Kit	BioVision	K778
G6p Kit	BioVision	K657
PK Activity Kit	BioVision	K709
HYP Kit	Sigma	MAK008
Maxima Cdna Kit	ThermoFisher	K1641
Sybr Green Qpcr Kit	NEB	M3003L
GSH Kit	Abcam	AB138881
AA Standards	Sigma	AAS18

Tgfβ	R&D systems	240-B
Dmem	Corning	10-013
Penn Strep	Corning	30-002CL
Glutamine	Corning	25-005-CL
Dasa-10	Millipore	550602
Tepp-46	MedChemExpress	HY-18657
TriZol	ThermoFisher	15596018
Taa	Fisher	AC424530250
Bleomycin	Selleck Chem	s1214
RNA Imax	Thermo	13778030
Siptbp1	Santa Cruz	sc-38280
Optimem	Thermo	11058021
10x Ripa	Millipore	20-188
Bradford	Biorad	5000201
Ecl	Thermo	32106
Trizol	Thermo	15596026
IHC-Tek DAB Peroxidase Substrate Kit	IHC World	IW-1600
Pitc	Thermo	26922
BS3	Thermo	21580
**PCR primers**		
Gene	Sequence (5'-3')	
**Human**		
hPHGDHF	GGAGGAGATCTGGCCTCTCT	
hPHDGHR	GTCATTCAGCAAGCCTGTCG	
hPSPHF	GGACTCCCTTTTAAGCAGATCTCA	
hPSPHR	TTCCCAGGGAGGTGAGCTG	
hPSAT1F	GCGGCCATGGAGAAGCTTAG	
hPSAT1R	ATGCCTCCCACAGACACGTA	
hSHMT1F	GTGACCACCACCACTCACAA	
hSHMT1R	ACAGCAACCCCTTTCCTGTAG	
hSHMT2F	GCTGCCCTAGACCAGAGTTG	
hSHMT2R	GCAGAGGCCGAGCCG	
hCOL1A1F	GGTCAGATGGGCCCCCG	
hCOL1A1R	GCACCATCATTTCCACGAGC	
hPKM2F	ATTATTTGAGGAACTCCGCCGCCT	
hPKM2R	ATTCCGGGTCACAGCAATGATGG	
hActinF	CTCGCCTTTGCCGATCC	
hActinR	TCTCCATGTCGTCCCAGTTG	
PKM-E9	CTTCTTATAAGTGTTTAGCAGCAGCT	
PKM-E10	GGGGCCATAATCGTCCTCACCA	
PKM-E11	CAGGTGGGCCTGACGAGCTG	

PKM-E5	CCTGTGGCTGGACTACAAGA	
PKM-E6	CCATATCAACATCCTGCTCGACC	

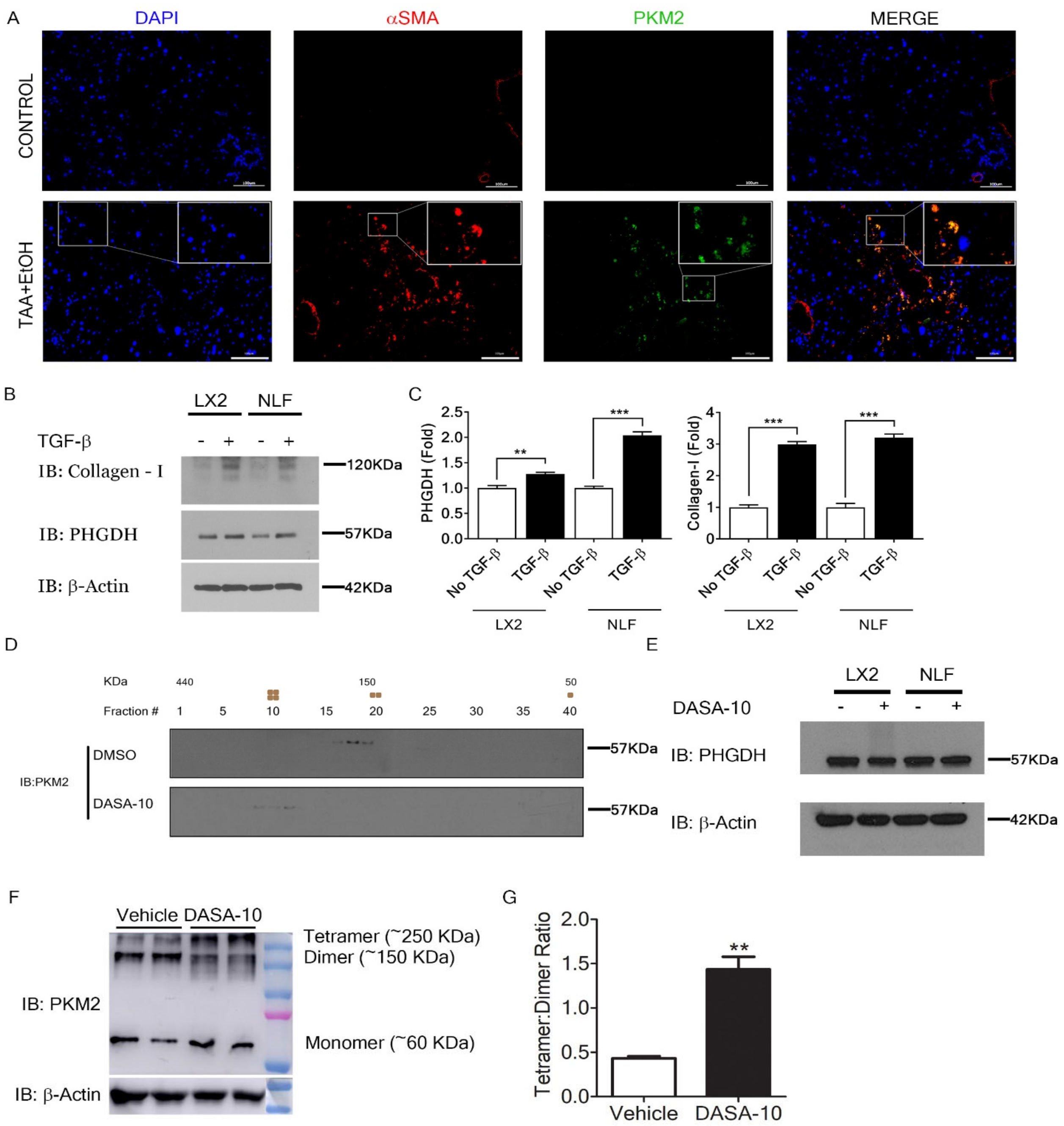

Supplementary figure S1 PKM2 is expressed in myofibroblasts.

(A) Representative images of IF co-staining α-SMA (red) with PKM2 (green) in liver sections from mice that were not treated (top panel) or induced liver fibrosis by TAA-alcohol (bottom panel). **(B) & (C)** Cellular levels of collagen (IB:collagen-1) and PHGDH (IB:PHGDH) in LX2 cells and human primary lung fibroblasts (NLF) with (+) or without (-) TGFβ treatment was

analyzed by immunoblot (B). Quantification of PHGDH (left) and collagen-1 (right) levels in LX2 cells and NFL with (TGFβ, black bar) or without (no TGFβ, open bar) TGFβ treatment (C). The PHGDH and collagen levels are presented as fold changes by comparing to that in cells without TGFβ treatment as reference as 1. **(D)** Immunoblot analyses of PKM2 (IB:PKM2) in chromatography fractions (fraction # is indicated at top of panel) of lysate of LX2 cells. The cells were treated with DASA-10 or DMSO following TGFβ treatment. The fractions equivalent to tetramer and dimer are indicated by symbols on top. **(E)** Cellular levels of PHGDH (IB: PHGDH) in LX2 cells and NLF with (+) or without (-) DASA-10 treatment following TGFβ treatment was analyzed by immunoblot. **(F)** Immunoblot analyses of PKM2 monomer, dimer, and tetramer in LX2 cell lysate. The cells were treated with DASA-10 or DMSO following TGFβ treatment. The cell lysates were subjected to crosslinking using BS3 before native electrophoresis. Most right are molecular weight markers. Monomer, dimer, and tetramer PKM2 are indicated on side. **(G)** Quantification of PKM2 dimer (~150KDa) to tetramer (~250KDa) shift was done using the ratio of band densities of the respective molecular weights. Immunoblots of β-actin (IB: β-actin) in (B), (E), and (F) are loading control. *MWs in B, D, and E are indicated on the right.* Error bars in C represent mean ± S.E.M.

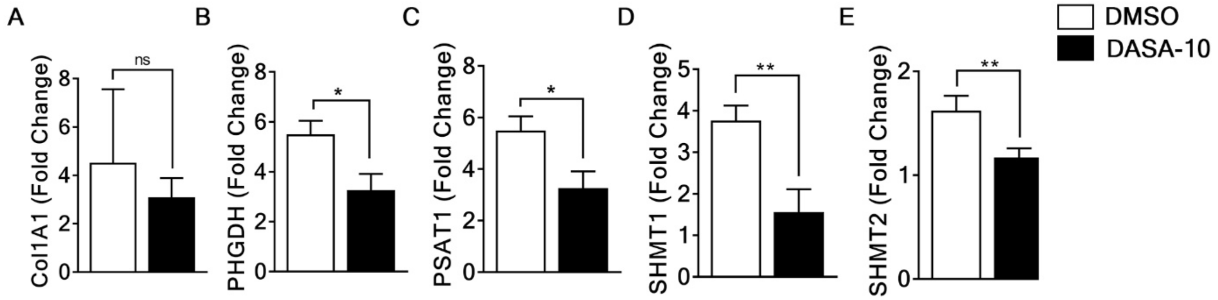

Supplementary figure S2 PKM2 activator DASA-10 decreases metabolic enzymes that are involved in serine/glycine metabolism.

Cellular levels of mRNA of collagen (Col1A1, A), PHGDH (B), PSAT1 (C), SHMT1 (D), and SHMT2 (E) in LX2 cells with DASA -10 (black bar) or DMSO (open bar) treatment following TGFβ treatment were analyzed by qRT-PCR. The cellular mRNA levels are presented as fold change by comparing controls. Error bars represent mean ± S.E.M.

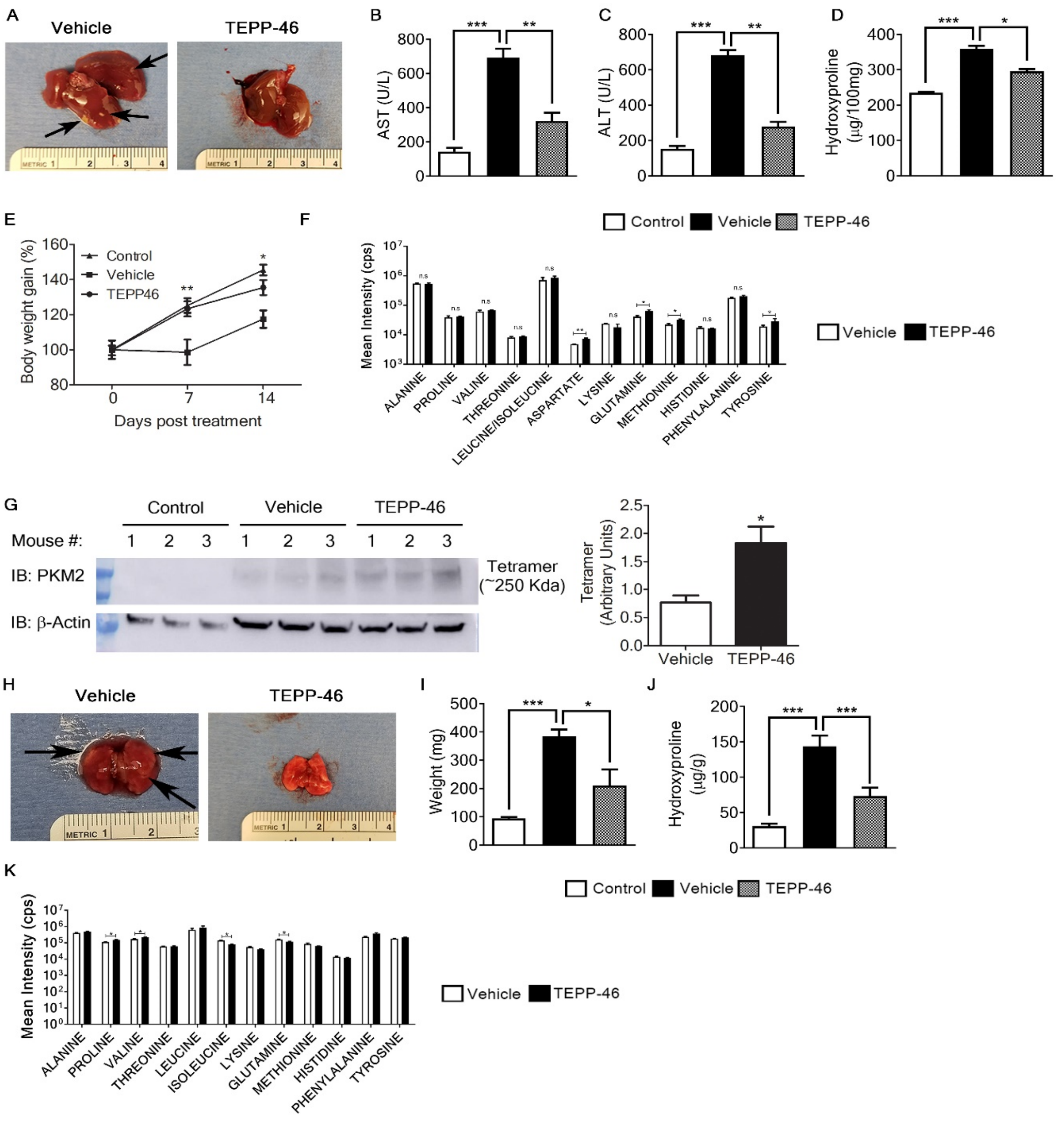

Supplementary figure S3 (A) & (G) Representative images of collected liver (A) and lung (G). Arrows indicate the fibrotic features. **(B)** & **(C)** Levels of serum AST (B) and ALT (C) in blood circulation of animals that were treated by the indicated agents were analyzed via commercial service (CPath). **(D) & (I)** Hydroxyproline in fibrotic liver (D) and fibrotic lung (I) of mice treated indicated agents. The hydroxyproline is presented as μg of hydroxyproline in lysate of per 100mg of fibrotic liver tissue or per gram of fibrotic lung tissue. **(E)** Body weights of the liver fibrotic

mice treated by indicated agents at the end point of experiments. **(G)** Liver lysates from control and treated animals were cross-linked using BS3 and subjected to immunoblot for PKM2 and tetramer formation was observed (left panel, most left is molecular weight markers) and the tetramer fraction (240KDa) band densities were quantified and represented as arbitrary units (A.U.). (Right panel). **(I)** Lung weights of the lung fibrosis mice treated TEPP46 (black bar) or vehicle (open bar) at the end point of experiments. **(F)** & **(K)** Quantitative analyses of amino acid levels in tissue extracts of fibrotic liver (F) and fibrotic lung (J) from mice treated with indicated agents by HPLC-MS. Error bars in B, C, D, E, F, G, H, I, J represent mean ± S.E.M.
